# Application of Wireless Magnetic Sensors in the Urban Environment and Their Accuracy Verification

**DOI:** 10.3390/s23125740

**Published:** 2023-06-20

**Authors:** Kristián Čulík, Vladimíra Štefancová, Karol Hrudkay

**Affiliations:** 1 University Science Park, University of Žilina, Univerzitná 1, 01026 Žilina, Slovakia; karol.hrudkay@uniza.sk; 2 Department of Railway Transport, Faculty of Operation and Economics of Transport and Communications, University of Žilina, Univerzitná 1, 01026 Žilina, Slovakia; vladimira.stefancova@uniza.sk

**Keywords:** sensors, city, verification, traffic survey, city transport, traffic

## Abstract

In a smart city, sensors are essential elements—the source of up-to-date traffic information. This article deals with magnetic sensors connected to wireless sensor networks (WSNs). They have a low investment cost, a long lifetime, and easy installation. However, it is still necessary to disturb the road surface locally during their installation. All road lanes to and from the city center of Žilina have sensors that send data at five-minute intervals. They send up-to-date information about the traffic flow’s intensity, speed, and composition. The LoRa network ensures the data transmission, but in the event of failure, the 4G/LTE modem realizes the backup transmission. The disadvantage of this application of sensors is their accuracy. The research task was to compare the outputs from the WSN with a traffic survey. The appropriate method for the traffic survey on the selected road profile is a video recording and speed measurement using the Sierzega radar. The results show distorted values, mainly for short intervals. The most accurate output from magnetic sensors is the number of vehicles. On the other hand, traffic flow composition and speed measurement are relatively inaccurate because it is not easy to identify vehicles based on dynamic length. Another problem with sensors is frequent communication outages, which cause an accumulation of values after the outage ends. The secondary objective of the paper is to describe the traffic sensor network and its publicly accessible database. In the end, there are several proposals for data usage.

## 1. Introduction

Vehicle detection in the transport system must provide accurate data. Better traffic information makes better decisions about traffic. The deployment of intelligent transport systems (ITS) requires high-quality input data. ITS combines various technologies, such as sensors, GPS, and communication networks, to enable real-time monitoring and control of transportation networks. Its goal is to improve mobility, enhance transportation safety, reduce traffic congestion, and minimize the negative environmental impact of transportation. Its applications include intelligent traffic management, electronic toll collection, advanced public transportation systems, intelligent parking systems, etc. In the current state of available technologies and the demands on transportation itself, the wireless sensor network is a beneficial technology that offers a solution for many applications and traffic management systems.

Sensors are used to monitor a change in some physical characteristic, which can also be widely used in transport. Some sensors monitor the speed of a vehicle, others the weight, the number of axles, or the occupancy of a parking space. Currently, there are many sensors on the market with the relevant technology, each of which has its own advantages and disadvantages. The choice of a particular type of sensor depends on the application’s conditions. Current vehicle detection and classification systems work on the principle of ultrasonic sensors, acoustic sensors [1], infrared sensors [2], inductive loops [3], magnetic sensors [4], video sensors [5], laser sensors [6], and microwave radars [7]. The authors in [8,9] divide sensors into invasive and non-invasive. Invasive sensors are those that interfere with the roadway or pavement. These include induction loops or magnetometers. They require the removal of the top layer of asphalt or concrete during their installation. Such installations are associated with potential failures—for example, when placing inductive loops in the area in front of an intersection. In such places, truck braking occurs, which causes the asphalt to ripple. Therefore, the induction loop may break and thus cease to function. The second type of detectors, called non-invasive, such as infrared radar, ultrasonic sensors, or video cameras, can be placed behind the roadside, i.e., they do not interfere with the road. Their performance may be affected by adverse weather conditions or even a lack of light [10,11]. Accuracy problems can also occur when too many vehicles are in the sensing range.

**Figure 1:**
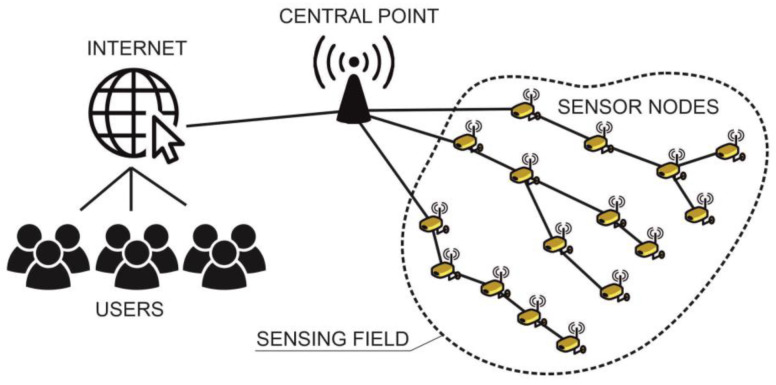
Scheme of a wireless sensor network.

Sensors in cities are connected to a wireless sensor network (WSN) [12]. WSN is a network of autonomous sensor nodes. They communicate wirelessly and collect and transmit data to a central location or server. These sensor nodes are small, low-cost devices with sensing, processing, and communication capabilities, enabling them to monitor and measure physical parameters. In traffic, they can measure, for example, the change in a magnetic field (the motion of vehicles). Figure 1 shows a schematic diagram of a WSN. In road transport, they monitor traffic in an assigned area using devices that measure physical traffic parameters such as density, volume, waiting time, communication throughput, pollution, and icing. Wireless sensor networks are becoming increasingly popular due to their flexibility in solving problems in various applications. They can enhance many areas of people’s lives. Sensor networks are used not only in transportation and road traffic, but they are also being applied in military, medical, or environmental domains [13,14].

According to [15], the use of magnetic sensors for vehicle detection in WSNs has the following advantages:

- No significant damage to the lane surface is required during installation.
- The magnetic sensor detects vehicles through changes in the earth’s magnetic field, so it cannot be easily affected by climate.
- The sensor does not react to non-ferromagnetic objects, which reduces its error rate.
- There is a possibility to categorize vehicles according to the type of magnetic field disturbance [16].

The problem with sensors can be their power supply. Powering sensors is problematic, especially if there are many of them over a large area of the city (e.g., lanes at all entrances and exits). In such cases, it is necessary to use sensors that can operate autonomously for at least several years [4].

The research in this paper deals primarily with magnetic sensors. These use the anisotropic magneto-resistive (AMR) principle to sense the vehicle [8]. Magnetic sensors have the advantages of reliability, accuracy, low cost, and robustness. In general, many types of battery-powered magnetic vehicle detectors are available. Their diameter is usually from 5 to 10 cm, and their height is from 5 to 15 cm. These dimensions make them practical and compact devices. Vehicle detection occurs based on a simple principle. The vehicle’s metal parts disturb the Earth’s magnetic field [17,18]. The Earth is a natural magnet with a surface magnetic field of about 500–600 mGs. The magnetic field remains relatively stable in a specific region. Due to the influence of the ferromagnetic substance represented by the metallic components of the vehicles, the magnetic field will be disturbed in a given area. A magnetic sensor is a kind of chip that can convert a magnetic signal into an electrical signal. Therefore, the sensor detects the vehicle based on a change in the magnetic field. Based on the duration of this change, the sensor can approximately determine the length of the car and thus classify it into the appropriate category.

Magnetometers based on the AMR principle can ensure vehicle classification, measure vehicle speed, and detect congestion [19,20]. The combination of sensor data acquisition and subsequent analysis via deep learning and the neural network is also advantageous [21,22].

**Figure 2:**
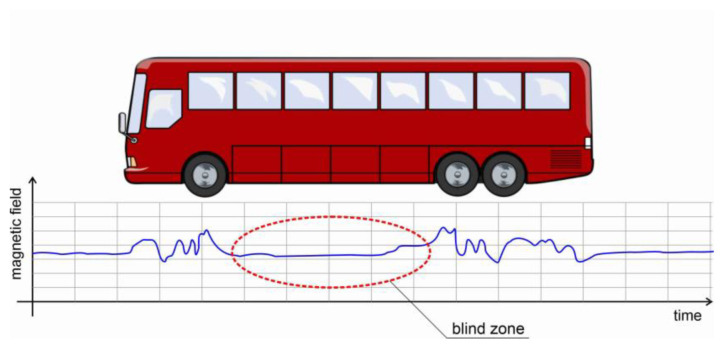
Example of blind zone of passing vehicle shown in the graph of the disturbance of the Earth’s magnetic field.

However, errors can also occur when detecting vehicles. There is a blind zone of geomagnetic signals between the front and rear axles of the vehicle. The blind zone is very noticeable, especially for large vehicles with high chassis, such as trucks, buses, SUVs, etc. It leads to problems with multiple detections, especially when moving at low speeds. Figure 2 shows the bus whose front and rear wheel areas have the strongest magnetic interference signal. However, between the front and rear axles there is the blind zone of the magnetic signal, which may cause the sensor to misjudge the vehicle identification.

At the same time, a non-standard position or movement of the vehicle when driving through the sensor area can be a problem. Sometimes, the location of sensors is before an intersection or a few meters before a pedestrian crossing. In such places, there is a high probability of the vehicle stopping. It is a reason why sensors do not detect the vehicle or detect it several times. It may occur when a car stops directly above or at the edge of the detector, which may disturb the traditional principle of operation of the magnetometer.

Smaller cars can also pose a problem for detection. If they move close to the edge, the sensor cannot detect them.

A very similar study comes from 2006 [23]. Authors Cheung, Ergen, and Varaiya investigated the accuracy of a wireless magnetic sensor network. Different from our solution, in which the sensor is always permanently embedded in the road, the authors from the USA had detectors glued to the pavement. The networks provide a detection rate of 98% and achieve 90% accuracy in average vehicle length and speed estimates. The localized change associated with the magnetic sensor allows the classification of the vehicles based on the magnetic signature with 80% accuracy. The research used the same methods as our study. They used video recording to find the number and type of vehicles. They even determined the speed through video analysis. 

The authors in [24] focused not on WSN but on clarification of vehicle detection in low signal-to-noise ratio (SNR) conditions and reliable vehicle speed calculation. With the proper algorithm, they calculate vehicle speed with a low average error rate. Study [25] also developed enhanced algorithms for improved outputs from magnetic sensors. It introduced a basic vehicle detection algorithm (DWVDA) with less computational complexity for vehicle counting in low-traffic volume conditions. The authors improved the detection performance in jam-flow conditions with a “tailgating effect” between the front and rear vehicles. They proposed an improved vehicle detection algorithm (SA-DWVDA) for traffic conditions. Magnetic sensors and inductive loops have similar characteristics.

According to [8], the magnetic sensor has some deviations compared to the inductive loops. However, it is an alternative solution with a low acquisition cost in traffic conditions with wide variations. In this study [8], the authors found significant relative differences between the outputs from these two detector types. Most of the differences were in the ±20% range. During the survey, the inductive loop detector reported 13,713 vehicles, while the magnetic sensor reported 13,407 vehicles, resulting in an overall difference of 306 vehicles (2.28%).

## 2. Magnetic Sensors in the City of Žilina

The sensory transport network in Žilina was created within the CleverNet project, supported by the EU Interreg V-A Slovak Republic “Implementation of innovative sensor networks in cross-border regions”. The project is co-financed by the European Regional Development Fund. The solver of the project was the University of Žilina. The partners of this project are Centrum dopravního výzkumu, v. v. i. CITIQ s. r. o. CityOne s. r. o. and UNIZA Technology Incubator s. r. o. [26]. 

### 2.1. Placement of Sensors

The mentioned network contains 26 sensors and two other sensors in front of the city office and the parking lot (near the University Science Park). They count the number of incoming and outgoing vehicles in the parking lot.

**Figure 3:**
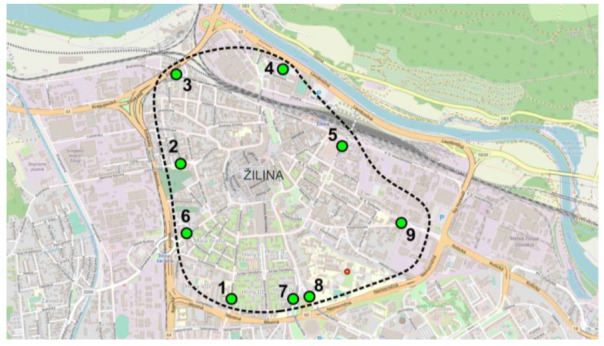
Geofencing and sensors on the city map. Streets: Komenského (1), Martina Rázusa (2), Bratislavská (3), Kysucká (4), 1. mája (5), Hálkova (6), Tajovského (7), Vysokoškolákov (8), and Košická (9).

These 26 sensors together form a virtual fence called “geofencing” around the city center (Figure 3). They are placed in the following streets: Komenského (1), Martina Rázusa (2), Bratislavská (3), Kysucká (4), 1. mája (5), Hálkova (6), Tajovského (7), Vysokoškolákov (8), and Košická (9). Traffic sensors monitor the number of vehicles, their length, and speed at all entrances and exits from the city center. It is thus possible to identify peak hours, i.e., the times of maximum vehicle presence in the city center. Obtained data helps design and modify parking policies, analyze pedestrian safety at pedestrian crossings, and serve other purposes.

Figure 3 shows that the detectors record almost all vehicles entering (IN) or exiting (OUT) the city center, which provides a picture of the traffic situation and the traffic load on the entrance/exit roads in the city. 

In their current configuration, the sensors do not cover every road that connects the city center to the surrounding areas. Drivers may also use various other, less important service roads. Additionally, the accuracy of the sensors is not 100%. Therefore, the traffic sensor network system does not record a certain proportion of the vehicles. 

The sensor traffic network in Žilina is maximally complex because every entrance/exit lane to/from the city center has a wireless magnetic detector. The city center is thus comprehensively closed. However, there are a few streets without sensors, but the intensity on them is very low.

The individual sensors within the network communicate with the central node (receiver) via the LoRaWAN (long-range wide-area network) [27,28]. It was necessary to choose a suitable IoT network. The basic requirements, in this case, were:

- coverage of the required area (approx. 5–6 km^2^) with several points (gateways),
- resistance to interference in the urban environment,
- independent dedicated network without ongoing data transfer fees,
- low energy consumption (battery life in sensors).

LoRa is best suited to these requirements. Another option was Sigfox, but it does not have coverage in Žilina. The narrowband technology of IQRF (technology for wireless packet-oriented communication via radio frequency) could suffer from potential transmission interference. 

Sensors also have other interfaces, but not for sending data. They serve to upgrade the sensor software (perhaps Bluetooth) or for experimental purposes. The Wi-Fi interface allows reading raw (unprocessed) data.

The LoRa network ensures the data transmission, but in the event of its failure, the backup transmission is realized via a 4G/LTE modem. Every 5 min, the sensor sends a number of counted vehicles up to 4096 (=212, 12-bit counter) and every 70 min, up to 4,294,967,296 (=232, 32-bit counter).

The locations of the network antennas are on the roofs of the Municipal Office in Žilina and the University Science Park of the University of Žilina. The sensors operate continuously 24 h a day and send collected data to the system every 5 min in small data packets [29,30]. 

Table 1 below shows the complete list of sensors connected to the network in Žilina. Each location contains 2 to 5 sensors, depending on the number of lanes. 

**Figure 4:**
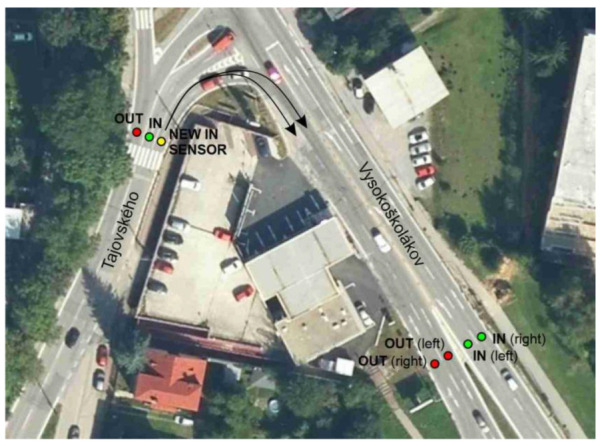
Location of sensors on Vysokoškolákov and Tajovského street. Source: processed from mapy.cz by authors.

Table 1 shows the street name with the sensors and their configuration, i.e., the number of lanes at the entrance and exit. Five out of the total number of locations have two sensors. It means the road section has one lane in both directions (left lane and right lane). In these locations, there is always one sensor to the city center (IN) and one from the city center (OUT). The remaining areas have more than one sensor installed due to the number of lanes. One of the network sensors, added in September 2021, removed a systematic error in vehicle counting. At the end of Tajovského Street, cars heading to the right were not detected (missing IN lane) but were detected at the exit (OUT). Figure 4 shows a representation of this new sensor on the map.

### 2.2. Technical Specification and Installation

**Figure 5:**
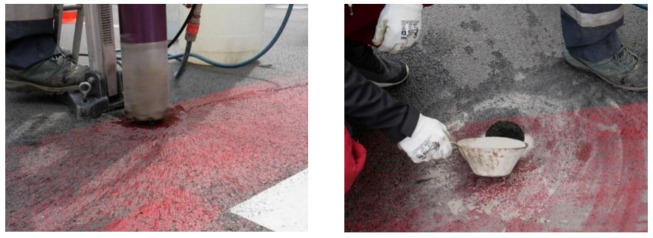
Installing the sensor—drilling the hole for the sensor (**left**) and sealing the hole after installation (**right**). Source: authors.

All sensors listed in Table 1 are magnetic without an external power supply, communicating wirelessly. In this case, the magnetometers are at a depth of 150 mm in the center of the lane. The location out of the vehicle wheel’s path ensures sensor durability. The placement of this device is relatively simple and does not require any significant interference from the road. The basis for mounting the detector is to drill a core hole in the roadway in the center of the lane with a diameter of approximately 100 mm (Figure 5, left). The detectors in Žilina are cylindrical, measuring 100 × 130 mm [29]. The sensor device is then placed in this hole and re-paved with a layer of asphalt. After installation, it is necessary to seal the hole on the road surface (Figure 5, right). Therefore, be easily identified from the outside.

The sensor acquisition costs are not high. In addition, the installation and operation are not demanding. Sensors do not require a connection to the electricity grid. Each sensor has a long-life battery. The expected lifetime of the device is three years. These attributes make this type of detector an ideal and simple device for traffic surveys within smaller towns or specific areas. The main advantages of traffic magnetometers are:

- Accuracy: Traffic magnetometers are highly accurate in measuring traffic volume and speed. They can distinguish between different types of vehicles and provide data in real time.
- Traffic magnetometers do not interfere with traffic flow or cause any disruption to road users.
- They are durable and require minimal maintenance. They are designed to withstand harsh weather conditions and can operate for a long time.
- Cost-effective solution compared to other traffic monitoring technologies.
- Traffic magnetometers can collect and transmit data online, making it easier for transportation agencies to analyze and make informed decisions about traffic management.

The sensor network in Žilina includes traffic magnetometers and sensors installed to measure road or pavement surface icing. These sensors are only in two places: in front of the Žilina City Hall and near the entrance to the University Science Park of the University of Žilina. However, the research in this article does not include them.

## 3. WSN and Its Database

The essence of the research contained in this paper is to verify the accuracy of sensor network data. The data described in the following lines is available in a public database. The principle of this research is to compare the downloaded data from the database with a traffic survey conducted in a specific location. The traffic survey used video recording. Then it was necessary to manually evaluate the video records to ensure the accuracy of the results.

### 3.1. Database of Sensor Network

Sensors send data to the system at intervals of at least 5 min. When collecting data, the detector can record the number of passing vehicles, their length, and their speed. According to these characteristics, the system divides vehicles into three groups in the database. This database is publicly accessible on the system operator’s website. It can be used, for example, to predict future transport requirements [26,31].

Based on length, the system categorizes vehicles into cars in the length interval of 3–7 m, smaller trucks such as vans in the length interval of 7–14 m, and trucks/buses/trolleybuses in the length interval of 14–30 m. If the sensor cannot detect the length of the vehicle, it will classify it as “uncategorized”. It can happen if the vehicle is too short or too long, or if there is a measurement error. However, there are only a few uncategorized vehicles out of the total number. Table 2 shows all vehicle categories.

Regarding vehicle speed, the database divides vehicles into three speed intervals. The first interval is the speed up to 30 km·h^−1^, the second is the interval from 30 km·h^−1^ to 60 km·h^−1^. The last group consists of vehicles that pass through the detector area at speeds above 60 km·h^−1^. The web interface also presents the percentage of speeding vehicles. The speed limit for this comparison is 60 km·h^−1^.

The database collects not only the latest data on traffic flows but also information from previous periods. It is possible to view data from any day or specific time interval since the start of operation or launch of the sensor traffic network.

The database has several functions and options for filtering data according to the user’s requirements. Filtering is possible according to the following criteria:

- Gate—It is possible to select any sensor area (from nine). It is also possible to select all gates at once.
- IN—The user can select one or more lanes heading to the city center.
- OUT—There is a possibility to choose one or more lanes leading out of the city center.
- Length category—It is possible to set a specific vehicle length category (cars, vans, trucks, and not categorized) or a combination of them.
- Speed category—The user can set a specific speed interval (<30 km·h^−1^; 30–60 km·h^−1^; >60 km·h^−1^), their combination, or select all.
- Group by time—It is possible to group data automatically or in intervals of 15 min, 30 min, 1 h, 6 h, 12 h, or 1 day.

In the upper-right part of the database, it is possible to select the time interval for data visualization. The user can set specific days, hours, exact minutes, and even seconds. However, the system may display inaccurate values when the user enters a short period in seconds or minutes. This error occurs because the sensors do not transmit data immediately after vehicle detection. 

Sensors send collected data at least every 5 min. Therefore, the longer the period the user chooses, the more accurate the result. The system records data from the start of the project. It is easy to compare any period in the records. 

**Figure 6:**
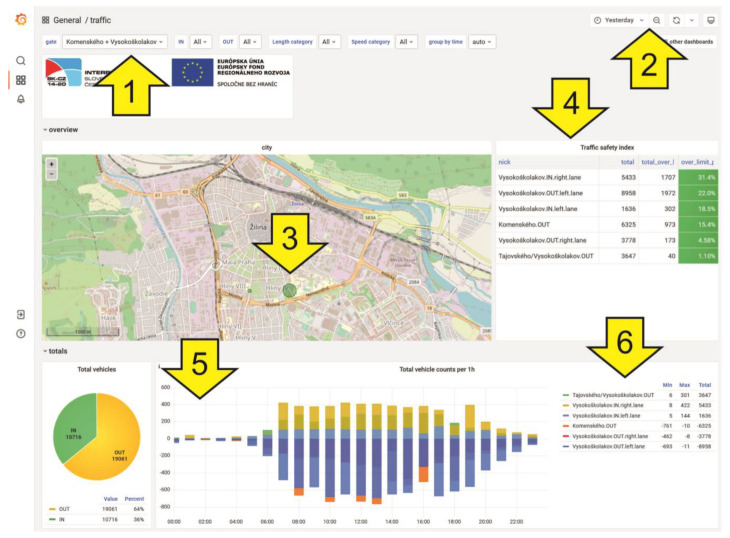
Grafana interface: (1) data filters, (2) time filter, (3) map visualization, (4) traffic safety index, (5) graphical data visualization, (6) tabular data visualization. Source: authors.

Figure 6 contains a description of the database user interface (Grafana) and the identification of controls. Grafana is a multi-platform open-source analytics and interactive visualization web application. It provides charts, graphs, and alerts for the web when connected to supported data sources.

### 3.2. Traffic Survey

This traffic survey was based on two devices, namely traffic detection and the use of video footage. The first device was a video-based survey camera, and the second device was a Sierzega SR4 traffic detection device. The Sierzega SR4 traffic counter is a device used to collect statistical data on road traffic. The used version of the radar device allows vehicle counting without affecting normal traffic flow. This device, powered by one or two 6V 12Ah batteries, has enough power to last approximately one week. The automatic traffic counter can measure parameters such as vehicle speed in km·h^−1^ in the range of 3–254 km·h^−1^ (with +/− 3% error), vehicle length in decimeters (with +/− 20% error), space between vehicles in seconds, and the time and date. The device senses the traffic flow in both lanes. The far lane may have biased results compared to the adjacent lane. The user can download the collected data from the device using a cable or wirelessly via Bluetooth technology [32].

Sierzega SR4 classifies the recorded vehicles into four categories, numbered 1 to 4. Category 1, according to the manual, represents motorcycles or vehicles with lengths up to 2.2 m. Category 2 includes standard passenger cars between 2.2 and 5.5 m long. Category 3 represents vehicles with lengths between 5.5 and 9.5 m. The last fourth category represents vehicles over 9.5 m [33]. 

Before the actual traffic survey, it was necessary to establish the measurement methodology, devices, and their correct locations. It is also necessary to choose suitable vehicle categories before the traffic survey. The sensors divide the vehicles into only three categories and the Sierzega measuring device into four. Nevertheless, we have divided the vehicles into nine groups to ensure a sorting of the nine categories into 4 or 3. The chosen vehicle categories were:

- passenger cars (OA), minibuses, and ambulances,
- buses (A),
- trolleybuses (Tr),
- light freight vehicles (LNV)—up to 3.5 tons—vans and trucks with one single rear axle,
- medium freight vehicles (SNV)—3.5 to 12 tons—trucks with two axles,
- heavy freight vehicles (TNV)—over 12 tons—with multiple rear axles + trailers,
- trucks with trailers (NS),
- motorcycles (M),
- bicycles (B).

The traffic survey was carried out at the location of the sensors on 1 May Street in two phases. The first of the surveys lasted 12 h. The survey lasted from 5:00 to 17:00 on 11 November 2021.

This traffic survey aimed to record the number of vehicles and their types. A responsible person could place the video camera in the parked car. This type of traffic survey does not require a high-quality video recording compared to vehicle license plate recordings. The correct position of the Sierzega SR4 device was approximately 20 m from a traffic sign. We have chosen a typical period of twelve hours.

**Figure 7:**
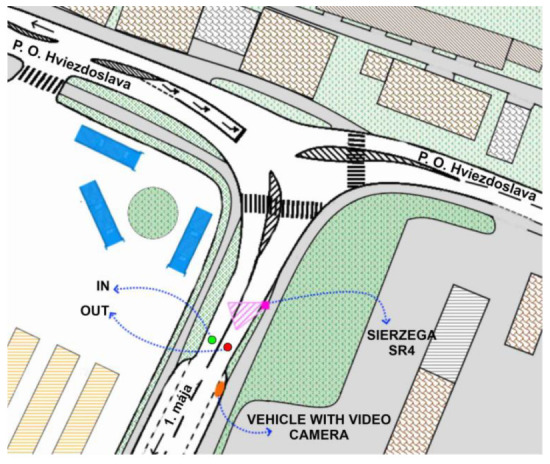
Traffic survey 11 November 2021 on 1. Mája Street—schematic representation. Source: authors.

Figure 7 shows the location of the investigated sensors on May Street, along with the intersection and other infrastructure. It also shows the placement of devices during the traffic survey, along with descriptions.

## 4. Results

The data from the Sierzega SR4 radar was exported using the manufacturer’s computer application as a text file and subsequently processed in Microsoft Excel 16.0. The video footage was analyzed by manually counting and categorizing the vehicles from the video footage into pre-prepared census forms at 15-min intervals.

### 4.1. Vehicle Categories

In the traffic survey, we divided vehicles into seven categories only. Motorcycles and bicycles were very rare during the survey. Therefore, we removed these two categories. There is also a low chance of their detection by the sensor. However, the traffic sensor network system categorizes vehicles into only three groups based on length: cars, vans, trucks, and “not categorized”.

The manual video analysis could not determine the exact dimensions of the vehicles. However, it is possible to expect that the category “cars” would include all passenger automobiles and light freight vehicles, most of which were vans or smaller trucks with a single-wheel rear axle. According to the technical specifications of several van manufacturers, the external length of this type of vehicle ranges from 6.3 to 6.9 m. 

All single-unit buses and trolleybuses of the public transport operator and the suburban bus transport operator in Žilina have a length of fewer than 14 m. Therefore, we can expect them in the ‘vans’ category. We can include medium and heavy freight vehicles in this category. The length of such vehicles will also not exceed 14 m. 

The last length category (trucks) will include all articulated public transport vehicles (articulated trolleybuses and articulated buses). According to the information on public transport fleets, these vehicles have an outer length between 17.59 m and 18.75 m [34]. The maximum permitted length of a truck with a trailer is 16.5 m in Slovakia. It means that we included these vehicles in the “truck” category.

Since the aim of the research was not to create advanced statistical analyses, we used only MS Excel software to evaluate the data. Outputs from WSN, or Grafana interfaces, are also obtained in CSV format, which is suitable for immediate import into this software. Right there, we created all the calculations and the comparison of the outputs from the WSN and the traffic survey.

### 4.2. Accurancy of Sensors

Table 3 and Table 4 show the accuracies of the sensors globally during the entire survey and the peak quarter hour, peak hour, lowest off-peak quarter hour, and lowest off-peak hour. Overall, the accuracy of the OUT sensor shows a relative difference of 8.32% compared to the actual numbers. This number was calculated according to Formula (1).

(1) $$SA=\frac{V_{TS}-V_{MS}}{V_{TS}}\cdot 100[\%]$$

where:

- SA—sensor accuracy (relative difference)
- V_TS_—number of vehicles counted by magnetic sensors.
- V_MS_—number of vehicles counted by magnetic sensors.

**Table 3:** Absolute and relative accuracy of the OUT sensor (unit: vehicles). Source: authors.

Description	Time Interval	Traffic Survey (Video)	Magnetic Sensor	Absolute Difference	Relative Difference
The entire survey	05:00–17:00	4616	4232	−384	8.32%
Peak quarter hour	16:00–16:15	149	96	−53	35.57%
Peak hour	13:45–14:45	519	462	−57	10.98%
Off-peak quarter hour	05:30–05:45	33	19	−14	42.42%
Off-peak hour	05:00–06:00	143	127	−16	11.19%

**Table 4:** Absolute and relative accuracy of the IN sensor (unit: vehicles). Source: authors.

Description	Time Interval	Traffic Survey (Video)	Magnetic Sensor	Absolute Difference	Relative Difference
The entire survey	05:00–17:00	4673	4440	−233	4.99%
Peak quarter hour	15:45–16:00	138	97	−41	29.71%
Peak hour	15:45–16:45	511	454	−57	11.15%
Off-peak quarter hour	05:00–05:15	13	9	−4	30.77%
Off-peak hour	05:00–6:00	116	100	−16	13.79%

Table 4 evaluates the accuracy of the IN sensor. The total deviation of the IN sensor was 4.99% for the 12-h duration of the traffic survey. The total number of vehicles leaving the city center was 4616. The number of vehicles in the entrance lane was 4673.

The OUT sensor detected a total of 384 vehicles, which is less than in reality. The IN sensor was more accurate and recorded an absolute difference of 233 vehicles. Both relative differences (8.32% and 4.99%) are quite acceptable. 

More significant deviations, observed in both sensors, occurred during maximum and minimum quarter hours. In these time intervals, the relative differences for both sensors ranged from 29.71% to 42.42%. In this case, the problem could be that the 15-min intervals are too short to be evaluated correctly. Data is sent to the database at least every 5 min. Sensors do not have synchronized data sending at five-minute times (xx:00, xx:05, xx:10, etc.). They only send a data packet every five minutes. Therefore, short time intervals are not accurate. For example, a sensor sent data at 3:56 p.m. Then it transmitted the next 4 min of vehicle counting in the next packet at 4:01 p.m. It means that the mentioned 4 min will not be included in the examined quarter of 3:45–4:00. For this reason, such large deviations could occur precisely at 15-min intervals. However, this short-term inaccuracy is not significant for the further use of data and forecasts.

### 4.3. Accurancy of Sensors during Whole Survey

**Figure 8:**
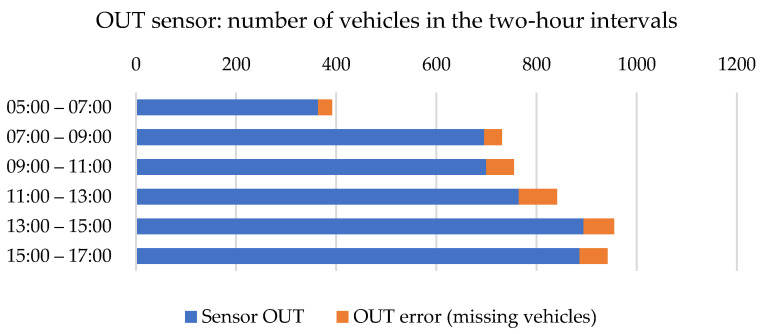
Numbers of vehicles counted by OUT sensor. Source: authors.

Figure 8 shows the diagram of the recorded vehicles on the OUT sensor divided into 2-h intervals during the entire survey. The numbers obtained from the sensor embedded in the road are blue. The orange color represents the number of missing vehicles compared to the traffic survey. The values measured by the sensor were 4.8 to 9.0% lower than the actual number of passing vehicles.

**Figure 9:**
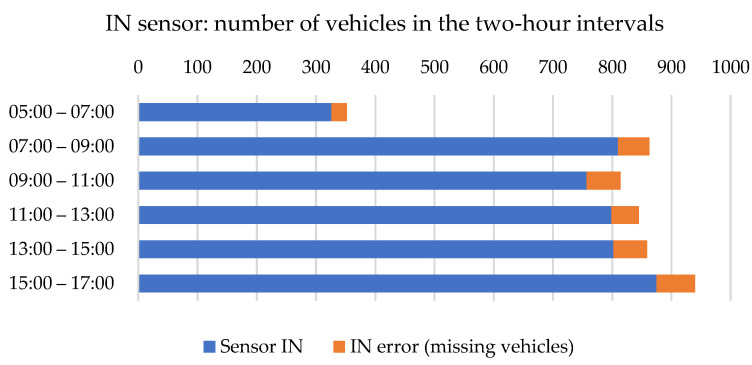
Numbers of vehicles counted by IN sensor. Source: authors.

Figure 9 shows the error rate of the IN sensor positioned in the opposite road lane. Similar to the sensor at the exit lane, it has only a negative deviation. It means the total number of vehicles in the 2-h interval was 5.4 to 7.4% lower than reality.

At a closer look, it is possible to say that the OUT sensor is slightly more inaccurate than the IN sensor, but the difference between them is minimal. Therefore, we can consider both sensors accurate and reliable. They sent data regularly and without errors during the whole traffic survey.

The measurement on 11 November 2022 had ideal weather conditions (dry road, sunny weather). In the next part of the research, it is necessary to check the accuracy in worse conditions that can affect transmitting, battery power, and vehicle detection.

Interestingly, both sensors always recorded fewer vehicles and never more than the actual numbers. It can mean that there were probably no multiple detections of vehicles, which is the most common error with this type of device.

### 4.4. Speed Measurement

The research subject was also accuracy verification. All sensors measure and record the vehicle’s speed. We used the Sierzega SR4 traffic radar during the traffic survey. The speed comparison is made for the entire survey without breaking it down into narrower time intervals. 

The sensors measure the speed according to the vehicle’s passage time, but it is not possible to extract the speed of each vehicle. The total numbers of cars in the three speed intervals are only available. Therefore, we divided the measured data from the radar into the same speed intervals.

The vehicle classification and counting are 100% reliable. In this part of the research, we cannot determine the exact reliability of the speed determined by the sensors. It is because the device, the Sierzega SR4, can also have a certain degree of deviation. Thus, the following tables show only the absolute difference between the numbers from the Sierzega SR4 device and the sensors. They also contain estimates of speed measurement reliability. The comparison for the OUT sensor is in Table 5. Table 6 shows the comparison for the sensor IN.

As can be seen from the tables, both sensors show significant differences. The IN sensor is more accurate than the OUT sensor. We compared the results with those of an external radar device. It was placed on the side of the road, near the IN lane. Our experience shows that the radar always more accurately measures the lane closer to the device. Vehicles in the far lane overlap more, so the accuracy of measuring their speeds is lower.

The smallest difference was recorded with the IN sensor in the speed interval from 0 to 30 km·h^−1^ for 53 vehicles. Such a discrepancy could be considered quite relevant. In addition, the difference in the same speed interval for the OUT sensor (147 vehicles) is relatively accurate if we regard the total number. The most striking differences can be observed in the remaining two speed intervals, when both sensors in the middle interval recorded significantly fewer vehicles compared to the Sierzega SR4. On the contrary, in the group of vehicles with a speed of more than 60 km·h^−1^, both sensors recorded a significantly higher number of vehicles compared to the radar. The OUT sensor recorded 491 speeding drivers (speeds more than 60 km·h^−1^), while the radar did not record anyone. In addition, the IN sensor recorded 430 of these vehicles, which is an incomparable difference. Both values (491 and 430) can be considered very unlikely due to the type of condition of the researched road section. The sensors on 1. Mája Street are located approximately 30 m before the intersection, and there is also a pedestrian crossing. Vehicles heading towards the center must turn at an angle of 90 degrees.

We estimated that most vehicles in this section will move at a speed between 30 km·h^−1^ and 60 km·h^−1^, which is confirmed by both sensors and radar.

At the end of this comparison, we can say that even though the data from the Sierzega SR4 radar cannot be considered accurate. Therefore, it is not even possible to explicitly determine the accuracy of the sensors in determining speeds. The comparison showed that the numbers for the first speed interval are relatively accurate, with differences of 147 and 53 vehicles (OUT and IN). It is a good result. Inconsistencies occur in the second and especially the third speed intervals. It means that the speed measurement by sensors at location 1 (Maja Street) is not accurate. It requires optimization.

In the case of rearranging data from sensors, moving the values from the last interval (above 60 km·h^−1^) to the middle one, the errors compared to the radar would be at the level of:

- +9.3% OUT < 30 km·h^−1^
−4.2% OUT (30–60) km·h^−1^
−4.9% IN < 30 km·h^−1^
−1.6% IN (30–60) km·h^−1^

### 4.5. Vehicle Categorization

The last part of the research was to determine the accuracy of the sensor network when classifying vehicles into individual categories. Not very satisfactory results were obtained in this category. The evaluation can be found in Table 7 for the OUT sensor and in Table 8 for the IN sensor.

Both sensors achieved similar inaccurate results. Both sensors show the lowest relative difference in the category ‘CARS’. Even this difference is significant and does not provide a relevant picture of the composition of the traffic flow in the given location. The ‘VANS’ and ‘TRUCKS’ categories saw even more significant differences. Therefore, we can say that sensors are not reliable for categorizing vehicles.

Low accuracy at very short intervals is mainly caused by problematic time synchronization. We carried out the traffic survey in real-time. However, the sensors in WSN send data at 5-min unsynchronized intervals. Therefore, the moment of receiving data can be at 10:00, 10:01, or 10:05. It means that one 15-min interval in the system can contain 1/3 of the counted vehicles that belong to another real-time quarter-hour.

The ‘CARS’ category includes all vehicles between 3 and 7 m long. It means that all cars and light trucks are in this group. Even if we counted all the vans and cars that passed through the place of the traffic survey, we still would not even get the number listed in the ‘CARS’ category. It is equally improbable that up to 615 vehicles longer than 9.5 m would leave the center during the survey.

Therefore, it is possible to conclude that even under ideal weather conditions, the sensors work very imprecisely when categorizing vehicles. It would be necessary to check them and possibly repair or optimize their data evaluation process.

## 5. Discussion

The traffic monitoring solution we described in this article is quite simple. It works on the principle of magnetic sensors, which send data wirelessly. Such a solution has several advantages and disadvantages compared to other traffic surveillance systems. The obvious benefits include:

- The magnetic sensors we used do not require electricity.
- The sensors are compact, so their installation requires only a small hole.
- The installation damages the road surface only minimally.
- Low acquisition costs compared to induction loops or systems using video detection.
- Data transfer during operation is free of charge.
- Resistance to interference in the urban environment.
- Low visibility (night or fog) does not affect detection.

Our solution has a relatively simple implementation in an urban environment, in which it is possible to cover approximately 5 to 6 km^2^ with such a network.

However, if we compare our application of sensors with modern systems, for example, video detection or automatic number plate recognition (ANPR), we find that:

- Magnetic sensors do not provide information about the direction of vehicles. The system cannot detect which gate the car entered the area through and which gate it left. In the case of scanning number plates, we know this information.
- The accuracy of classification based on dynamic length is very low. During more than two years of operation of the sensors, we have found that vehicles are often classified incorrectly. In an urban environment, there are always at least 90% passenger cars, but despite this, the sensors often give a much lower number.
- The speed measurement accuracy is also low. For some applications, such data may be insufficient.

A much more modern and accurate traffic monitoring system is automatic video evaluation. The authors in [35] describe a traffic surveillance system for obtaining comprehensive vehicle information, including type, number of axles, speed, length, current driving lane, and traffic volume. They use instance segmentation implemented by Mask R-CNN. Mask R-CNN (mask region convolutional neural network) is a deep learning model that combines the concepts of object detection and instance segmentation. It is an extension of the faster R-CNN (region-based convolutional neural network) model widely used for object detection. However, compared to magnetic sensors, there may be a problem with detection at night or during fog. The inaccuracy of vehicle detection from the video in [35] was lower than 5%. According to [36], recent advances in technology have made roadside surveillance not only based on cameras but also on other emerging technologies aimed at achieving various aims and objectives. Researchers have been most interested in the development and applications of radio tomographic imaging (RTI), wireless sensor networks (WSNs), and computer vision for roadside surveillance. Radio tomographic imaging is an emerging technology that localizes and tracks moving physical objects in an area surrounded by simple and inexpensive radios in wireless networks [37].

More than two years have passed since the installation of magnetic sensors at all entrances to the city center of Žilina. However, during this period, the data generated and published by the system were not verified. The research described in this article is the first step toward revealing the accuracy of the data. The sensor network provides the relevant outputs, such as total vehicle numbers and speed categories. The data appears to be approximately accurate and valid for future applications. The categorization of vehicles is much more imprecise. Magnetic sensors have relatively big problems. We recommend distributing these numbers proportionally between the individual categories according to constant shares.

An important aspect is the data usage and applications that could work with data from the sensor traffic network. The following lines describe the possibilities of using online information about the traffic flow in the city of Žilina [38].

### Data Usage and Future Research

One of the characteristics that the magnetic traffic sensors in the city of Žilina within the sensory transport network can record is the speed of identified vehicles, or speed interval. This data could serve the police force. The police can observe and evaluate the vehicle speed at every city center entrance and exit [39]. The sensors undoubtedly achieve certain inaccuracies. However, they provide a good overview of the speed ratios in individual sections.

Some road sections, covered by magnetic sensors, connect light-controlled intersections. Such locations are on these streets: Komenského, Hálkova, Košická, 1. Mája, Vysokoškolákov. Individual sensors send collected vehicle data to the sensor traffic network database every 5 min. This frequently updated traffic information can be used to optimize the management of traffic lights to reduce traffic congestion [34]. Once the sensors detect the presence of a vehicle, they send the data to a central control system that analyzes the data and decides which traffic lights to activate. The control system can also consider factors such as the time of day, the flow of traffic, and the presence of pedestrians. If multiple vehicles are detected from different directions, the control system can use an algorithm to determine the best sequence for activating the traffic lights to minimize delays and congestion [40].

The traffic sensor network performs continuous traffic surveys. A proper traffic survey is a time-, financial-, and human-intensive activity that usually covers only a short time. The sensory traffic network could be an authoritative indicator of the development of traffic flow. The detectors within the described wireless network do not provide very detailed data. The vehicle classification includes only three categories. In addition, it ensures inaccurate results and does not show the relevant traffic flow composition. These indicative values can be helpful for the city administration to organize a regular traffic survey to verify these data, or they can be an indicator of the emergence of a traffic problem in the city in the future. In simpler terms, the traffic sensors within the sensor network represent the constant monitoring of traffic development in the city territory. Thanks to them, we can forecast traffic problems in future periods.

Since the sensory transport network covers all main entrances or exits to the city center, it is possible to track when most vehicles flow into the center and how long they stay there. Only number plate scanning can identify each car at the entrance and exit. Therefore, it can determine the exact delay time inside the city center.

Information about the possibilities, methods, and amount of parking spaces in the city center of Žilina is known. There are often problems with parking inside the city. The number of cars grows, but the number of parking spaces remains the same. However, many Eastern European cities have problems with parking for several reasons. Žilina, similar to many other cities, has a historic city center with narrow streets not designed for car parking. The second reason is that the municipality experienced rapid urbanization during the 20th century without adequate planning for infrastructure, including parking. The car is also considered a symbol of well-being. It is leading to a high demand for parking spaces.

The construction of new parking spaces is a limited solution. It has significant financial costs, especially the territorial limitation, which does not allow the continuous construction of parking areas in the future. Of course, it is possible to build underground garages under the new buildings, but even this may not be the decisive aspect that can solve future parking problems in the city.

The ideal solution could be prevention, which would encourage drivers to reduce the need to use a car for transport to the city center and motivate them to use another type of transport. This can reduce the number of vehicles entering the center and partially reduce the number of missing parking spaces. To achieve this goal, it would be possible to try to apply a dynamic parking policy. In such a system, the prices of parking in the city center are calculated according to the number of vehicles entering the center during the day, which would be recorded by the sensory traffic network. Rate changes could occur weekly, always based on the evaluation of data from the previous week. Future rates would be available in advance, for example, on the city’s website, so that drivers can decide in advance whether it is worthwhile for them to use a private car or choose another mode of transport. 

However, such a solution is not easily applicable. Another pillar would be requested in the form of building temporary parking lots on the outskirts of the city, from where drivers would use an alternative mode of transport. This would prevent increased parking directly in the city. Of course, such a solution is not so easily applicable, as it would require another pillar, for example, in the form of building temporary parking lots on the outskirts of the city, from where drivers would already use an alternative mode of transport and thus avoid increased parking inside the city. However, such a solution is not easily applicable. Another pillar would be requested in the form of building temporary parking lots on the outskirts of the city, from where drivers would use an alternative mode of transport. This would prevent increased parking directly in the city.

## 6. Conclusions

This article aimed to verify the accuracy of the sensory transport network in the city of Žilina through a traffic survey. We subsequently analyzed and compared the traffic survey results with data from sensors installed under the road surface.

For verification, we conducted a 12-h traffic survey at selected sensor locations on 11 November 2021. We used a camera, which continuously recorded the traffic during the survey. This type of traffic survey is accurate and easy for manual evaluation. The location on May 1st Street was chosen because there are only two sensors—one in the IN direction and the other in the OUT direction. There is also a relatively flat section here, thanks to which the Sierzega radar could better measure the speed of incoming and outgoing vehicles. There is a relatively high intensity of traffic in the area of the traffic survey. Furthermore, many urban and suburban public transport lines run here due to the location next to the bus station.

In this paper, we found that our sensors count vehicles with an accuracy of 95% (IN sensor) and 92% (OUT sensor). These results are not satisfactory, but they are unbiased. We can compare them with an older study that achieved much greater accuracy 18 years ago. The authors in [23] provided a two-hour trace of measurements with magnetic traffic sensors. A total of 793 vehicles were observed. The correct detection rate of the sensor network is 98%, compared with 86% for the inductive loop. Moreover, for shorter time intervals, we achieved even more imprecise values.

When analyzing and verifying the data, we compared the accuracy of the sensors individually and together during the entire survey and during peak and lowest off-peak times. We also measured the sensors’ accuracy in determining the speeds of passing vehicles and in vehicle categorization. Some results that emerged from the verification were satisfactory—traffic sensors can be considered reliable (for vehicle counting), but some were less accurate (speeds and categorization).

One of the possibilities for working with data in the future is to recalculate the number of vehicles into individual categories. The main disadvantage of magnetic sensors application in Žilina is their imprecise vehicle classification. In many cases, it turned out that the number of medium-long vehicles (vans) was higher than the number of vehicles up to 7 m (cars). The sensor’s calibration can improve its accuracy. Another problem is that several sensors were destroyed during the railway reconstruction and adjacent roads.

This article describes only one of the pillars of research in this area. During almost two years of collecting data from WSN, we discovered fundamental errors in data transmission. The transmission outage lasts from several minutes to several days. At the end of this outage, all the data accumulates into one total number. If we want a histogram without blank and extreme columns, we must divide the total number into all empty 15-min intervals. Data correction consisted of multi-factorial regression analysis, which considered:

- day of the week,
- day of the year (period),
- level of COVID-19 measures (during the data collection, there was an impact of the COVID-19 pandemic on traffic),
- type of day (working day, weekend, holiday),
- school holidays, etc.

The mentioned procedure can correct the missing values at 15-min intervals. Only after this correction was it possible to forecast future values. For this forecasting, we are currently creating a neural network. In this way, we can simulate the expected load on the urban transport network from the mentioned factors in the following periods.

Predicting traffic flow is a complex task due to the influence of many factors, such as historical traffic data, weather conditions, events, road construction, and real-time traffic information. Artificial intelligence (AI) techniques, such as machine learning and deep learning, can be used to develop predictive models that analyze these factors and make accurate predictions about traffic flow. Machine learning algorithms can be trained on historical traffic data to learn patterns and relationships between variables such as time of day, day of the week, weather conditions, and traffic volume. These models can forecast future traffic flow based on the current inputs. AI can analyze real-time data from traffic sensors and process it. Then AI algorithms can detect traffic patterns, identify congestion points, and predict traffic flow.

## Figures and Tables

**Table 1:** List of sensors used in city of Žilina and their configuration.

No.	Street Name	Number of Sensors	Configuration
1	Komenského	2	IN + OUT
2	Martin Rázusa	2	IN + OUT
3	Bratislavská	2	IN + OUT
4	Kysucká	2	IN + OUT
5	1. mája	2	IN + OUT
6	Hálkova	4	2 × IN + 2 × OUT
7	Tajovského	2	IN + OUT
8	Vysokoškolákov	4 + 1 ^1^	2 × IN + 2 × OUT
9	Košická	5	3 × IN + 2 × OUT

^1^ An additional a sensor described in the text.

**Table 2:** Vehicle classification and examples of vehicles in each category.

Category	Length (m)	Examples
Car	3–7	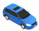
Van	7–14	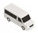
Truck	14–30	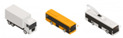
not categorized	–	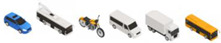

**Table 5:** Comparison of the OUT sensor and the Sierzega SR4 device for determining vehicle speeds. Source: authors.

Speed Interval	<30 km·h^−1^	30–60 km·h^−1^	>60 km·h^−1^
Sierzega SR4	1587	3050	0
Sensor OUT	1440	2431	491
Absolute difference	−147	−619	491
Relative difference	9.3%	20.3%	*

* Infinite/extreme difference.

**Table 6:** Comparison of the IN sensor and the Sierzega SR4 device for determining vehicle speeds. Source: authors.

Speed Interval	<30 km·h^−1^	30–60 km·h^−1^	>60 km·h^−1^
Sierzega SR4	1086	3250	4
Sensor IN	1139	2875	430
Absolute difference	53	−375	426
Relative difference	−4.9%	11.5%	*

* Infinite/extreme difference.

**Table 7:** Vehicles in each category according to OUT sensor and traffic survey. Source: authors.

Speed Interval	Cars	Vans	Trucks	Not Categ.
Traffic survey	3996	523	97	0
Sensor OUT	1673	2074	615	95
Absolute difference	−2323	+1551	+518	+95

**Table 8:** Vehicles in each category according to IN sensor and traffic survey. Source: authors.

Speed Interval	Cars	Vans	Trucks	Not Categ.
Traffic survey	4407	167	99	0
Sensor IN	2070	2115	259	91
Absolute difference	−2337	+1948	+160	+91

## Data Availability

Data are available on request due to restrictions on transport enterprises.
